# Unipolar and Bipolar Depressed Inpatients: correlations with Vitamin D and Cognitive Symptoms

**DOI:** 10.1192/j.eurpsy.2024.1131

**Published:** 2024-08-27

**Authors:** I. Bonfitto, G. Moniello, G. Francavilla, A. Bellomo, S. Dimalta

**Affiliations:** ^1^Department of Mental Health, ASL FG, Foggia; ^2^Department of Geriatric Medicine and Gerontology, ASL CE, Caserta; ^3^Department of Clinical and Experimental Medicine, University of Foggia, Foggia, Italy

## Abstract

**Introduction:**

Cognitive symptoms are the main factor of discomfort in depressed patients, persisting even during clinical remission (Conradi et al. Psychol Med 2011;41:1165-74) and inevitably compromising their quality of life (Fehnel et al. CNS Spectr 2013;25:1-10). Several studies have suggested the neuroprotective role of vitamin D, both through actions on the genome and rapid non-genomic mechanisms; so, low serum vitamin D levels are related to poorer cognitive performance (Goodwill et al. JAGS 2017;2:1-8), depressive disorders (Kjærgaard et al. Psyc Res 2011;190:221-225) and suicide risk (Umhau et al. PLoS One 2013;8:e51543).

**Objectives:**

to investigate relationships between serum vitamin D levels, depressive symptoms and cognitive performance in unipolar and bipolar depressed adults hospitalized for Major Depressive Episode (MDE).

**Methods:**

80 patients (34 M and 46 F; average age 48,96±14,17 years; 40% with bipolar depression) were examined. Depression was investigated using Hamilton Rating Scale for Depression (HAM-D), while cognitive functions were explored by: Rey Auditory Verbal Learning Test (RAVLT) and Rey-Osterrieth Complex Figure (ROCF) to assess verbal and visuospatial memory, respectively; Trail Making Test (TMT) and Stroop Color and Word Test to assess attention, spatial planning and cognitive flexibility. Venous blood sampling was used to determine serum Vitamin D levels (average level 15,67 ± 8,7 ng/ml).

**Results:**

At first, the serum level of vitamin D was found to be inversely correlated with HAM-D scores (p=0,0079), so that lower concentrations of vitamin D is related to greater severity of depression. In addiction, there were strongly significative positive correlations between low vitamin D levels and poorer RAVLT and ROCF scores and strongly significative negative correlations between vitamin serum level and higher scores in TMT and STROOP test, so that calcidiol deficit is associated with poor cognitive performance. Similarly, patients with higher HAM-D scores were found to have a greater cognitive impairment (lower RAVLT e ROCF scores and higher TMT e STROOP scores).

**Conclusions:**

In accordance with previous works, our study supports the close relationship between serum vitamin D levels and depressive morbidity. During MDE hypovitaminosis D is related to worse disease indices, such as severity of affective symptoms and cognitive impairment, without substantial differences between clinical manifestations of unipolar and bipolar depression, both in terms of affective and cognitive symptoms and disease severity. Considering that cognitive deficits are truly disabling because they may resist to common antidepressant treatments and, as a result, persist during stages of clinical remission, vitamin d supplementation, by minimizing cognitive dysfunction, could be a good strategy to reduce the risk of relapses and to improve patients’ functioning and quality of life.

**Disclosure of Interest:**

None Declared

